# Risk Factors for Psychiatric Disorders in Patients with Multiple Sclerosis—A Single-Center Study in the Polish Population

**DOI:** 10.3390/medicina60030376

**Published:** 2024-02-23

**Authors:** Hubert Mado, Michał Błachut, Anna Szczegielniak, Krzysztof Świerzy, Magdalena Zając, Katarzyna Kubicka-Bączyk, Piotr Gorczyca, Monika Adamczyk-Sowa

**Affiliations:** 1 Department of Neurology, Faculty of Medical Sciences in Zabrze, Medical University of Silesia, 40-752 Katowice, Poland; hib333@interia.pl (K.K.-B.); m.adamczyk.sowa@gmail.com (M.A.-S.); 2 Chair and Clinical Department of Psychiatry, Faculty of Medical Sciences in Zabrze, Medical University of Silesia, 40-752 Katowice, Poland; mblachut@sum.edu.pl (M.B.); pgorczyca@sum.edu.pl (P.G.); 3 Psychiatric Ward, Multi-Specialist District Hospital, 42-612 Tarnowskie Góry, Polandkafswierzy@gmail.com (K.Ś.); zajacmagdalenaa@gmail.com (M.Z.); 4 Department of Psychiatric Rehabilitation, Department of Psychiatry and Psychotherapy, Faculty of Medical Sciences in Katowice, Medical University of Silesia, 40-752 Katowice, Poland

**Keywords:** multiple sclerosis, comorbidity, mental disorders, neurology, psychiatry

## Abstract

*Aim*: The aim of this study was to determine the prevalence of mental disorders in a group of patients with multiple sclerosis (MS) during outpatient treatment. Additionally, an attempt was made to assess the influence of parameters related to patients and their clinical status on the prevalence of mental disorders. *Materials and Methods*: This study was conducted between 2017 and 2018 in a group of 103 patients with MS who underwent treatment at the Outpatient Clinic of Neurology at the Clinical Hospital No. 1 in Zabrze, Poland. Sociodemographic data were collected, and the course of the underlying disease and comorbidities underwent assessment. The Mini International Neuropsychiatric Interview (MINI) and psychiatric examination were used to assess the occurrence of mental disorders. *Results*: In this study, female subjects accounted for 67.96% of patients (mean age: 43 years). Of all patients, 67% of subjects were clinically diagnosed with mental disorders during their lifetime. The results of the MINI Questionnaire showed that 33% of MS patients had a history of a major depressive episode, while 8.7% of patients met the criteria for a depressive episode. The same number of patients were treated for recurrent depressive disorders. Generalized anxiety disorder was diagnosed in 10.7% of patients, agoraphobia in 8.7% and panic disorder in 7.8%. Most patients (94.2%) had a low risk of suicide, according to the MINI Questionnaire. This study did not show a significant influence of age, sex, duration of MS symptoms or severity of symptoms as expressed by the Expanded Disability Status Score (EDSS) on the prevalence of mental disorders (*p* = 0.05). However, a significantly higher median EDSS score was found in patients with a history of mental disorders (*p* = 0.03). Additionally, a significant negative correlation was found between having a family and a psychiatric diagnosis (*p* = 0.01). A statistically significant negative correlation was found between the level of education and the suicide risk as assessed by the MINI Questionnaire (*p* = 0.03). *Conclusions*: This study showed a high prevalence of mental disorders in patients with MS, of which depressive episodes and anxiety disorders were the most commonly reported. There may exist a relationship between the degree of disability of MS patients and a higher prevalence of mental disorders. Patients with MS who do not have a family may be more susceptible to mental disorders. In turn, patients with a lower level of education may show a higher risk of suicide. This suggests the need for psychological and psychiatric support for patients with MS, with particular consideration given to those who are alone, those with more severe disability and patients with a lower level of education.

## 1. Introduction

Multiple sclerosis (MS) is one of the more common autoimmune diseases, affecting approximately 2 to 3 million people worldwide [1]. The geographic distribution of patients with MS is not homogeneous and varies considerably from region to region. However, higher latitude is associated with a higher MS incidence, although there are exceptions to this rule [2,3]. As most affected individuals are in their early adulthood, MS has a significant impact on quality of life and is one of the most common causes of non-traumatic neurological disability in this age group in Europe and North America [4]. The course of the disease is heterogeneous; hence, different degrees of disease severity can be observed [5,6]. The moment of diagnosis is a stressful, life-changing event for most patients and their families. Therefore, the recognition of the emotional burden and the risk of developing mental disorders of differing intensity and severity, as well as adequate support, are of crucial importance for improving quality of life and compliance in the treatment of the underlying disease [7,8].

In the course of MS, various mood, behavioral and cognitive disorders may also occur. Some of them are interpreted as a consequence of brain damage resulting from the demyelination of axons or treatment-related adverse effects [9], while others are considered diverse psychological reactions to the progressive course of the disease, which leads to disability [10,11]. The pattern of their development is still under debate. However, it is believed that these various etiological factors often coexist [12,13]. Occasionally, patients in whom mental disorders are the first symptom of MS are also reported, which usually results in a delay in neurological diagnosis [14,15,16]. The first study describing the co-occurrence of mental disorders in patients with MS was conducted as early as the 1920s [17]. It was demonstrated that depressive episode symptoms occurred in 24–50% of MS patients, which is significantly more prevalent compared to the general population and most other neurological disorders [13,18,19,20,21]. In addition, a higher rate of suicide is observed in the group of MS patients [22]. The prevalence of anxiety disorders in the course of MS is estimated at 21–36% [13,23,24]. Other less common disorders include bipolar affective disorders, psychotic disorders and personality disorders [13,25]. Although mental disorders manifest more frequently in patients with MS than in the general population, they are often undiagnosed in clinical settings [26,27]. Mental disorders have an additional negative impact on the severity of the disability. As a result, the main elements associated with the co-occurrence of mental disorders in MS patients include a significantly lower quality of life and poorer patient compliance in the treatment process [28,29].

This is the first study in Poland whose aim was to determine the prevalence of mental disorders in a group of patients with MS treated on an outpatient basis and to assess the impact of sex, age, duration and severity of MS symptoms on the occurrence of mental disorders.

## 2. Materials and Methods

### 2.1. Characteristics of the Study Group

A total of 103 patients (70 women and 33 men) with MS diagnosed using the current McDonald criteria were enrolled in this study (age range: 18–66 years) [30,31].

All patients were recruited between January and December 2017 during treatment at the Outpatient Clinic at the Department of Neurology, Medical University of Silesia, Clinical Hospital No. 1 in Zabrze. The collection of all information from patients in the form of interviews and questionnaires was conducted in 2017–2018. The inclusion criteria were as follows: written informed consent to participate in the study, an age range of 18–70 years and MS diagnosed based on the current McDonald criteria [30,31]. The exclusion criteria were as follows: withdrawal of informed consent or refusal to participate in the study, disability score > 8.0 on the Kurtzke Expanded Disability Status Scale (EDSS) and cognitive impairment that prevented adequate understanding of the study. The approval of the Bioethics Committee of the Medical University of Silesia was obtained (no. KNW/022/KB1/136/16).

In the study group, basic sociodemographic data were collected (age, sex, level of education, duration and treatment of neurological symptoms and mental disorders, number of neurological and psychiatric hospitalizations and pharmacological treatment at the time of the study). At the same time, patients were examined by a neurologist to assess their disability status using the EDSS [32]. In the next stage, patients who were enrolled in the study were referred to psychiatrists for a psychiatric evaluation using the MINI International Neuropsychiatric Interview Questionnaire (version 6.0), which allows for the assessment of 15 most common mental and behavioral disorders included in DSM-IV and ICD-10 [33,34,35]. Another part of this diagnostic tool allowed for the assessment of the risk of suicide in the study group. The prevalence of each mental disorder during a lifetime was assessed during the medical examination and using the MINI Questionnaire.

### 2.2. The First Statistical Analysis

After a thorough psychiatric evaluation, the study group was divided into two subgroups, i.e., patients with and without mental disorders. Both groups were compared in terms of age, sex, disease duration and MS symptom severity according to the EDSS score to assess the possibility of the influence of these variables on the prevalence of mental disorders.

Statistical analysis was performed in the R environment version 3.3.2 (https://cran-archive.r-project.org/bin/windows/base/old/3.3.2/, accessed on 15 January 2024). The data obtained from the measurement were expressed as the arithmetic mean ± standard deviation and quartiles, while the data obtained by counting were expressed as percentages. Pearson χ2 test with Yates’ correction for continuity was performed for the univariate qualitative analysis. Student’s *t*-test was used to compare patient groups in terms of the results of normal distribution and equal variances, whereas the Welch test was used when the null hypothesis in the Fisher’s test for variance was rejected. The Mann–Whitney U test was performed for the results without normal distribution. A *p* < 0.05 was considered significant.

### 2.3. The Second Statistical Analysis

Using the scripting language R version 3.6.3 (https://www.r-project.org/, accessed on 15 January 2024), another independent statistical analysis was performed for a more in-depth analysis of the data, and a more thorough verification of the results was obtained. For the purpose of the analysis, patients with 6–11 lesions on T2-weighted images were included in the group of patients with 11–20 lesions.

#### 2.3.1. Correlations

To analyze the relationship between variables expressed on a nominal scale (dichotomous variables can adopt two variants (e.g., yes/no, women/men)), the Fi (ϕ) correlation coefficient was calculated. When the relationship between the pairs of variables on an interval scale (numerical, e.g., age) and on a nominal scale (dichotomous variables) was examined, the point-double series correlation coefficient was calculated. Spearman correlation was performed for pairs of variables on the interval and ordinal scales and the pairs of variables on the ordinal scale (both variables were expressed on this scale). The statistical significance of the obtained correlation coefficients was also tested. A significance level of 0.05 was adopted.

#### 2.3.2. Confirmatory Data Analysis (CDA)

The chi-square test was used to verify the research hypotheses on the presence of significantly statistical differences in data distribution for pairs of variables on the nominal scale (at least one variable adopts more than two variants (e.g., unmarried, married, single)). When the Cochran conditions were not met, Fisher’s exact test was used. The ANOVA test was used for the pairs of variables on interval and nominal scales (non-dichotomous variables). The Kruskal–Wallis test was used if the assumptions of normality and homogeneity of variance were not met. Because the group of patients with schizophrenia was too small (2 subjects), this group was removed from the analysis.

Outlier points were removed before analyses were performed:

1. In the case of the analysis of the correlation between the duration of MS treatment and the diagnosis of mental disorders at the time of the assessment, patients with anxiety disorders for whom the duration of MS treatment was 16 and 30 years were excluded;
2. In the case of the analysis of the correlation between the number of neurological hospitalizations and the diagnosis of mental disorders at the time of the assessment, the following patients were excluded: subjects with no disorders who underwent 10, 15 and 23 neurological hospitalizations; patients with adjustment disorders who indicated 50 neurological hospitalizations; subjects with anxiety disorders who underwent 10 and 13 neurological hospitalizations; those with depression who indicated 12 neurological hospitalizations.

A significance level of 0.05 was adopted.

## 3. Results

### 3.1. The First Statistical Analysis

In total, 103 patients were enrolled in this study, including 70 (67.96%) women and 33 (32.03%) men (mean age: 43 years; SD = 12.05). The duration of MS treatment ranged from less than one year to 30 years (mean duration: 5.9; SD = 5.64), and the duration of MS symptoms was up to 40 years (mean duration: 10.85; SD = 8.75). The EDSS score ranged from 0.5 to 8.0 (mean score: 3.2; SD = 1.61). Relapsing–remitting MS was the most prevalent form and was found in 89 (86.40%) patients, primary-progressive MS was reported in 3 (2.91%) patients and secondary-progressive MS was observed in 11 (10.67%) patients. In total, 97 (94.17%) subjects presented with more than 20 areas of demyelination on MRI. The number of neurological hospitalizations in this group ranged from 1 to 50 (mean: 4.74; SD = 7.53). In total, 56 (54.36%) patients were also treated for another somatic disease. Five (4.85%) patients had completed primary education, twenty-one (20.38%) vocational school, thirty-seven (35.92%) high school and forty (38.83%) patients had university education. Fifty-five (53.39%) patients declared to be professionally active at the time of the assessment (Table 1).

In patients who participated in this study and were assessed using the MINI Questionnaire, a history of a major depression episode was found in 33% of cases, while 8.7% met the criteria for a depressive episode at the time of the assessment. The same number of patients was treated for recurrent depressive disorders. A past manic/hypomanic episode affected 3.9% of patients, and bipolar affective disorders affected 2.9% of subjects. None of the subjects met the criteria for a current manic/hypomanic episode nor did they present with a history of mood disorders with psychotic symptoms. Among the current mental disorders, generalized anxiety disorder (GAD) was the most prevalent (10.7%), followed by agoraphobia (8.7%), panic disorder (7.8%), social phobia (4.9%), obsessive-compulsive disorder (2.9%) and post-traumatic stress disorder (PTSD) (1.9%). In the case of other mental and behavioral disorders, three patients presented with a history of a psychotic disorder, a current psychotic disorder and alcohol abuse, while two other patients from this group were diagnosed and treated for bulimia. Alcohol/substance abuse, anorexia, antisocial personality or intellectual disability were not reported (Table 2). 

**Figure 1:**
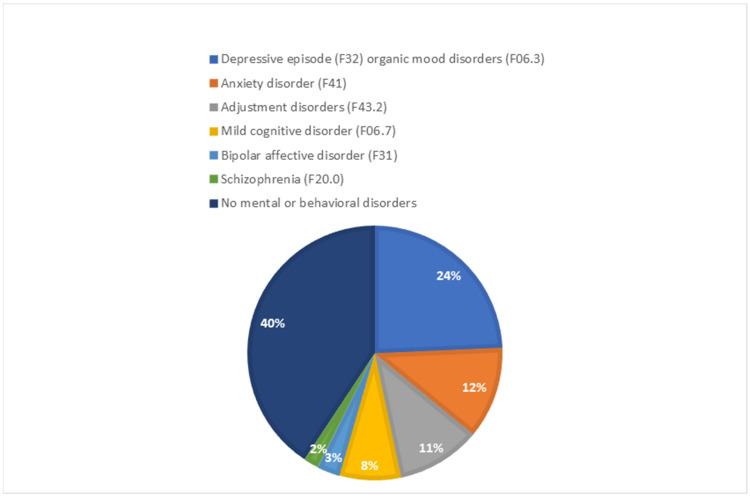
Current or past mental disorders in patients with multiple sclerosis based on psychiatric assessment.

Most patients (94.2%) had a low risk of suicide at the time of assessment using the MINI Questionnaire, while 1.9% and 3.9% of patients had a medium and high risk of suicide, respectively. The clinical psychiatric examination (psychiatric assessment without the MINI Questionnaire) showed current or past mental disorders in 70 patients (67.9%). The most common mental disorders diagnosed based on the International Classification of Diseases (ICD-10) included depressive episodes (F32) and organic mood disorders (F06.3), which were found in 24.3% of patients, followed by anxiety disorders (F41) (11.7%) and adjustment disorders (F43.2) in 10.7% of subjects. In addition, mild cognitive disorders (F06.7) (7.8%), bipolar affective disorders (F31) (2.9%) and schizophrenia (F20.0) (1.9%) were also observed (Figure 1).

There was no significant influence of age, sex, duration of MS symptoms or severity of disease symptoms measured by the EDSS scores on the presence of mental disorders in the MINI Questionnaire or the disorders that were present during the psychiatric assessment (*p* = 0.05). However, patients who presented with a history of mental disorders had significantly higher median EDSS scores (*p* = 0.03; rGlass −0.26). This could indicate a relationship between the severity of MS symptoms as measured by the EDSS scores and the occurrence of mental disorders at some point in life. 

### 3.2. The Second Statistical Analysis

#### 3.2.1. Descriptive Statistics

Risk of Suicide Using the MINI Questionnaire

Psychiatric Diagnosis at the Time of the Assessment

Clinically Confirmed Mental Disorders Occurring during the Lifetime of Patients (not Only during the Assessment)

Clinically relevant statistics for the points are presented in the form of tables in the Appendix A.

#### 3.2.2. Confirmatory Data Analysis (CDA)

##### The Assessment of the Risk of Suicide Using the MINI Questionnaire

Table 3 shows that the value of Spearman’s correlation coefficient for education and the risk of suicide reported during the study was statistically significant (*p* < 0.05). The negative sign of the coefficient indicates changes in opposite directions, which means that the risk of suicide was lower in patients with higher education. Table 4 shows descriptive statistics that reflect this relationship. Additionally, Table 5 shows the distribution of the data for this pair of variables.

##### Clinically Confirmed Psychiatric Diagnoses at the Time of the Assessment

Statistically significant differences in the distribution of data were found for the correlation between having a family and clinically confirmed psychiatric diagnosis at the time of the assessment. As shown in Table 6, not confirming any psychiatric diagnosis at the time of the assessment was observed more frequently in patients who had a family. Table 7 shows the *p*-values of the tests.

##### Clinically Diagnosed Mental Disorders during the Lifetime of Patients

The results of the correlations between clinically diagnosed mental disorders during the lifetime of patients and the specified variables indicated no statistically significant differences. All *p*-values were above a significance level of 0.05. The detailed results of the analyses are given in Table 8.

## 4. Discussion

In this study, we assessed the prevalence of mental disorders in a group of MS patients treated on an outpatient basis. Compared to similar studies related to this subject, our study group was selected in appropriate proportions in terms of sex, which was significant [35,36,37,38].

The results confirmed the reports of a frequent co-occurrence of mental disorders with MS [13,19,20,21,37,39,40]. According to the MINI Questionnaire, 33% of patients had a history of a depressive episode, while 8% had a depressive episode at the time of the assessment. Among anxiety disorders, GAD was the most prevalent (10.7%), followed by agoraphobia (8.7%) and panic disorder (7.8%), which is in line with other studies [25,36,39,41,42]. In total, 24% of patients were diagnosed with a depressive episode (F32) and organic mood disorders (F06.3) prior to or at the time of the assessment.

We also showed that, at the time of the clinical assessment, psychiatrists confirmed the occurrence of current or past mental disorders in 67% of patients with MS. These results are in line with those obtained by Galeazzi et al. [39]. Depressive disorders with symptoms meeting the criteria for a depressive episode and organic mood disorders were diagnosed in 24% of patients, anxiety disorders in 11.7% and adjustment disorders in 10.7%, whereas cognitive disorders were diagnosed in 7.8% of patients. Similar results were obtained by Marrie et al. [36], who compared diagnosing mood disorders in a group of MS patients using questionnaire methods and medical examination. Medical examination showed depressive disorders in 28% of patients and anxiety disorders in 11%. Using the questionnaires, those authors confirmed depression in 33% and anxiety disorders in 18% of patients [36]. 

Similar results were found in terms of the percentages of disorders using specific methods when our study was compared with the study by Marrie et al. [36]. Furthermore, in both cases, a significant discrepancy of the results was reported in terms of depressive episodes between the MINI Questionnaire and the psychiatric diagnosis. In our study, a slightly higher percentage may result from considering past and current episodes. 

Many studies assessing the occurrence of depressive disorders in MS patients are conflicting. Galeazzi et al. [39] assessed 50 patients with relapsing–remitting MS and found that 46% of patients presented with a major depressive disorder in their lifetime, 12% presented with a current major depressive episode, 6% had bipolar affective disorders and 10% were diagnosed with dysthymic disorder. Anxiety disorders were diagnosed in 36% of patients, with a simple phobia being the most prevalent (12%). Gottberg et al. demonstrated the occurrence of depressive disorders in 19% of MS patients [37]. Amtmann et al. [43] evaluated 166 patients with MS and found a depressive episode in 29% of patients. Karimi et al. [38] demonstrated depressive disorders in 47% of patients and anxiety disorders in 39% of MS patients. Feinstein et al. [44] analyzed 100 patients with MS, and their results showed that 17% of subjects met the criteria for the diagnosis of a major depressive episode. Korostil and Feinstein [45,46] evaluated 140 patients using the SCID-I questionnaire to diagnose anxiety disorders. The lifetime prevalence of any anxiety disorder was found in 35.7% of patients, generalized anxiety was reported in 18.6% of subjects, panic disorder in 10% and obsessive-compulsive disorder in 8.6% of the study group. De Cerqueira et al. [25] evaluated 60 patients with MS using the MINI Questionnaire. They found that 18.3% of subjects had a history of a depressive episode, 18.3% of patients were diagnosed with depression at the time of the assessment and 13.3% had a bipolar affective disorder. Among anxiety disorders, GAD was the most prevalent and was diagnosed in 16.7% of subjects, followed by panic disorders in 3.3% [25]. 

We used a very similar protocol and research tools. Our study also confirmed that mood and anxiety disorders were the most prevalent. However, we reported a lower percentage of bipolar affective disorders (3.9%) and a higher prevalence of panic disorders (7.8%). The literature shows that the possibility of the occurrence of mental disorders during the diagnosis and treatment of a chronic somatic disease with a high risk of disability is affected by disease-related stress, changes in daily functioning, the presence and the quality of social support, treatment-related adverse effects (e.g., steroids and other drugs), pathological structural processes (atrophy, damage) and central nervous system (CNS) dysfunction [9,10,11,12,39,40,46]. The simultaneous influence of these factors of varying severity on MS patients may account for the differences in the results published worldwide.

Attention should be paid to the group of 54% of patients who were simultaneously treated for other somatic comorbidities, which may influence the severity of mental disorders and may increase their prevalence, as indicated in the literature [47]. Nevertheless, our study did not show a statistically significant correlation between the presence of comorbidities and increased rates of any mental disorder. 

Over 95% of patients were diagnosed with multiple demyelinating lesions in the CNS. This is a very important etiological factor that can initiate, exacerbate or sustain the existence of mental disorders (mostly cognitive), as shown by Feinstein et al. [46]. However, our study did not show a statistically significant correlation between the number of lesions on T2-weighted images and mental disorders. 

The literature indicates that other possible factors for the co-occurrence of mental disorders with MS include adjustment difficulties associated with limitations, symptoms and social problems associated with MS, genetic susceptibility, structural brain abnormalities, an association between depression and immune and inflammatory changes and pharmacotherapy causing mental disorders, as well as personality features that predispose to the occurrence of mental disorders and diseases [13,48]. In terms of pharmacotherapy, Gasim et al. showed no statistically significant correlation between the use of disease-modifying therapy (DMT) and the occurrence of any adverse effects in the form of mental or behavioral disorders [49]. However, our study did not evaluate such a correlation. 

Interestingly, we found a statistically significant negative correlation between having a family and mental disorders at the time of the assessment. This fact supports the idea that MS patients who have a family may be less susceptible to mental disorders. This phenomenon could be explained by the support given to MS patients related to many spheres of life, including mental health. This phenomenon is not surprising, as it is widely believed that support from others can have a positive impact on mental and physical health [50]. However, we did not demonstrate a similar statistically significant correlation in terms of current or past mental disorders. 

In our study, we observed a statistically significant negative correlation between the level of education and the risk of suicide as assessed by the MINI Questionnaire. Patients with a higher level of education showed a lower risk of suicide. These results are in line with Karimi et al. [38], who reported the same relationship between the severity of depression and education. Karimi et al. also showed a statistically significant negative correlation between the severity of depression and economic status. However, we did not show such a correlation.

We demonstrated a statistically significant correlation between the median EDSS score and the occurrence of clinically diagnosed mental disorders during a lifetime. However, there was no statistical significance between the EDSS score and the occurrence of mental disorders during a lifetime. The result was very close to the significance level (*p* = 0.05), and the increase in the group size may have yielded clearer results. It seems that *p* could have obtained a value below 0.05. Therefore, it seems reasonable to assume a potential association between higher EDSS scores (which reflect a higher level of disability) and a higher prevalence of mental disorders. Similar results were presented by Sarısoy et al., who demonstrated that MS patients with higher EDSS scores showed more severe symptoms of depression [51]. Those authors also stressed a higher prevalence of other mental disorders in patients with MS, the severity of which also increased with higher EDSS scores. These mental disorders included somatization disorders, the occurrence of obsessive thoughts and activities, increased interpersonal sensitivity, aggressive behaviors, phobias, low self-esteem and eating disorders [51]. However, Janssens et al. [52] reported that MS patients and their partners showed high levels of anxiety and distress in the first years after diagnosis, but no statistically significant correlation between this finding and increased disability was found. 

In our study, current or past cognitive disorders were clinically diagnosed in only 7.8% of patients with MS. Other studies showed that cognitive impairment occurred in 43–70% of patients [53,54], which is a significant discrepancy. In our study, it may be related to the lack of additional tools and tests to detect cognitive impairment apart from obtaining a history and physical examination, which is one of the important limitations of this study. Reports analyzing the prevalence of mental disorders in MS based only on medical examination are scarce, and their results correspond to our findings [36]. Marrie et al. stressed the problem with diagnosing the real number of MS patients affected by depression or anxiety disorders [36]. Diagnostic methods do not improve the detection rates of these disorders. This may be due to the similarity of some symptoms or their overlap, as in the case of mental/motor retardation, malaise and anxiety. Additionally, the existing cognitive impairment causes further problems related to contact with patients.

The literature indicates that there is over a 2-fold higher risk of suicide in MS patients compared to the general population, and this risk is particularly high within the first year after the diagnosis of MS [52]. However, in our study, the risk of suicide as assessed by the MINI Questionnaire was low in most patients, medium in 1.9% of subjects and high in 3.9% of patients. These results are relatively lower than those published by Sarisoy et al. and Feinstein [51,55]. In those studies, 8.3% and 6.4% of patients had a history of a suicide attempt, and 8.3% showed a risk of suicide at the time of the assessment. In the above studies, all patients with a risk of suicide at the time of the assessment presented with the symptoms of depression when their medical history was taken [51,55]. These differences may be caused by a higher number of patients with progressive forms of MS who were enrolled in the study as compared to our study. Patients with suicidal tendencies were significantly more often alone, unsupported, had a family history of mental diseases, more social problems and comorbid episodes of depression, anxiety disorders or comorbid depression and anxiety disorders [8,40,55,56]. However, these are the features typical of a higher risk of suicide not only in the group of MS patients but in all patients. However, as previously mentioned, our study demonstrated a statistically significant negative correlation between the level of education of MS patients and the risk of suicide. 

According to a WHO report, 27% of the respondents in European Union countries experienced at least one mental disorder within a year [57]. In our study, the occurrence of current or past mental disorders was found in 67% of patients with MS, which indicates the significance of the problem in this group of patients. In their epidemiological study conducted in Poland in 2015, Kiejna et al. [58] showed that a major depressive disorder was present in 3% of subjects. It was significantly more prevalent in women (4%) than in men (1.9%). These data could be compared with our results. We found that 33% of MS patients had a history of a depressive episode or were diagnosed with such an episode at the time of the assessment. Such a comparison shows the enormous scale of mental disorders in the group of MS patients. Furthermore, these data suggest the need for psychological assistance for MS patients, particularly those with more severe disability, those without a family and those with a lower level of education. 

Patients with MS have numerous psychological challenges. It is well known that depression, fatigue and cognitive impairment are intertwined in MS. Fatigue is a crucial and exceedingly common symptom in the course of MS, and it is an urgent problem in the mental health matter of MS patients. In fact, this symptom affects motor function, sleep patterns, anxiety, depression and, consequently, overall quality of life in the course of this disease [59]. Fatigue has been shown to be a key aspect of overall quality of life in the course of MS [60]. Appropriate therapy for this condition is therefore extremely important. Physical activity, cognitive-behavioral therapy and pharmacotherapy have been shown to be beneficial in this regard [59]. Work capacity is also significantly impaired due to fatigue in the course of MS [60]. Work capacity is also affected by the cognitive impairment, emotional distress and heat sensitivity experienced due to the condition. In addition to fatigue, other important predictive factors are age, gender, EDSS score and education level [60]. Apart from the psychological problems classified in the International Statistical Classification of Diseases and Related Health Problems (ICD-10), it should be noted that patients also experience clinically known problems such as “chronic sorrow” as well as “MS euphoria”, where the subject shows cheerfulness and happiness but at the same time ignorance of the dysfunction and hardship in the course of the disease [61,62]. It is therefore important to quickly identify such patients and provide psychological help promptly. 

In a study by Johansson et al., the occurrence of symptoms of mood disorders and depression was shown to be variable over a 2-year period in the course of MS, and during that time half of the patients experienced depressive symptoms at least once [63]. This indicates that it is of crucial importance to develop strategies to identify these symptoms in MS patients and consequently to implement appropriate psychological and medical assistance, which could result in a definite improvement in these patients’ quality of life [63]. In addition, a study by Binzer et al. found that patients who have MS and depression have a significantly increased risk of worsening disability [64]. This finding highlights the importance of diagnosing depression in patients with MS as soon as possible, as well as implementing appropriate treatment [64]. Given the prevalence of depressive and anxiety disorders in the course of MS, it seems reasonable to consider the administration of selective serotonin reuptake inhibitors (SSRIs) in these patients. It was shown that, due to the improvement in mental status, appropriate monitoring but also adequate treatment of psychiatric symptoms can improve the long-term prognosis of the disease [65]. Moreover, studies are underway on the effect of SSRIs on the pathogenesis of MS. Indeed, serotonin has been shown to modulate the Th-17 immune response in the course of this condition [66]. Thus, it can be speculated that, abstracting from the very important role of SSRIs in improving mental status, they may exhibit strict effects targeting MS pathogenesis. This even creates a rationale for considering therapy with serotonergic drugs as an additional therapy for relapsing–remitting MS [66].

The most important limitations of this study include the size of the group and the lack of detailed assessment of the severity of depressive and cognitive disorders. Patients with the exacerbation of their underlying disease were not excluded from the study, which may have influenced the number of patients with mental disorders. In addition, the assessment of suicide risk based on the MINI Questionnaire does not seem to be completely reliable, although the results of a systematic review were not known before the completion of the study [67]. The lack of the specified “inter-rater reliability” for psychiatrists who clinically assessed patients is another limitation of this study. 

## 5. Conclusions

This study found that mental disorders were common in MS patients. Depressive and anxiety disorders were prevalent, which indicates the need for a systematic assessment of the mental status of MS patients. We did not confirm a significant influence of age, sex, duration or severity of MS symptoms on the occurrence of mental disorders in our group. However, this study showed that a higher degree of disability in MS patients, as measured by the EDSS score, may be associated with a higher prevalence of mental disorders. Furthermore, it was shown that a psychiatric diagnosis was made significantly more often in patients who had no family. Furthermore, patients with a lower level of education showed a higher risk of suicide, as assessed by the MINI Questionnaire. This suggests the need for psychological and psychiatric care for MS patients. The analysis of the literature and this study confirm the need for a detailed diagnosis of the co-occurring problems of MS patients to establish an optimal personalized treatment plan.

## Figures and Tables

**Table 1:** Baseline clinical data of MS patients.

	Number of Patients	Mean	Standard Deviation	Median	Min	Max
Age	103	43.07	12.05	43	18	66
Duration of MS treatment (years)	103	5.92	5.64	4	0	30
Duration of MS symptoms (years)	103	10.85	8.75	8	0	40
Disability (EDSS)	103	3.2	1.61	3	0.5	8
Number of neurological hospitalizations	103	4.74	7.53	2	1	50

**Table 2:** Mental disorders in patients with multiple sclerosis as determined by the MINI International Neuropsychiatric Interview.

Mental Disorders	No.	%
Current major depressive disorder	9	8.7
Past major depressive disorder	34	33.0
Current manic/hypomanic episode	0	0
Past manic/hypomanic episode	4	3.9
Panic disorder	8	7.8
Agoraphobia	9	8.7
Social phobia	5	4.9
Obsessive-compulsive disorder	3	2.9
PTSD	2	1.9
Alcohol abuse	0	0
Harmful use of alcohol	1	1.0
Use of other psychoactive substances	0	0
Harmful use of other psychoactive substances	0	0
Psychotic disorder	1	1
Past psychotic disorder	1	1
Current mood disorders with psychotic symptoms	1	1
Past mood disorders with psychotic symptoms	0	0
Anorexia	0	0
Bulimia	2	1.9
Generalized anxiety disorder	11	10.7
Antisocial personality	0	0
Recurrent depressive disorder	9	8.7
Bipolar affective disorder	3	2.9
Mental retardation	0	0

**Table 3:** *p*-values of the tests and values of the coefficients of correlations between the risk of suicide at the time of the assessment using the MINI Questionnaire and the specified variables.

Test	Variables	*p*	Value of the Coefficient of the Correlation
Spearman correlation	Age	0.62	0.05
Spearman correlation	Treatment duration	0.9	−0.01
Spearman correlation	Symptom duration	0.31	0.1
Spearman correlation	EDSS score	0.23	0.12
Spearman correlation	Number of neurological hospitalizations	0.41	0.08
Spearman correlation	Education	**0.03**	−0.21
Spearman correlation	Number of lesions on T2-weighted images	0.53	0.06
Fisher	Sex	0.28	
Fisher	Marital status	0.13	
Fisher	Livelihood	0.52	
Fisher	Family	0.81	
Fisher	Living with a family/alone	0.59	
Fisher	Comorbidities	0.29	
Fisher	Accidents and injuries	1	
Fisher	Form of MS	0.1	

**Table 4:** Descriptive statistics of the risk of suicide at the time of the assessment using the MINI Questionnaire based on education and years of schooling.

Risk of Suicide at the Time of the Assessment	*n*	Min	Max	Median
Low	97	0	3	2
Medium	2	2	2	2
High	4	0	2	1

0—elementary education (≤8 years); 1—vocational education (≤12 years); 2—secondary education (≤12 years); 3—higher education (≤18 years).

**Table 5:** Distribution of data for education and the risk of suicide at the time of the assessment using the MINI Questionnaire.

	Elementary		Vocational		Secondary		Higher	
	*n*	*n* [%]	*n*	*n* [%]	*n*	*n* [%]	*n*	*n* [%]
Low	4	80	19	90.48	34	91.89	40	100
Medium	0	0	0	0	2	5.41	0	0
High	1	20	2	9.52	1	2.7	0	0

**Table 6:** Distribution of psychiatric diagnoses at the time of the clinical assessment based on having a family.

	No		Yes	
	*n*	*n* [%]	*n*	*n* [%]
Not found	10	31.25	32	46.38
Adjustment disorders (F43.2)	6	18.75	5	7.25
Anxiety disorder (F41)	8	25	4	5.8
Depression (F32/F06)	4	12.5	21	30.43
Cognitive disorder (F06.7)	3	9.38	5	7.25
Bipolar affective disorder (F31)	1	3.12	2	2.9

**Table 7:** *p*-values of the tests evaluating correlations between clinically diagnosed mental disorders at the time of the assessment and the specified variables.

Test	Variables	*p*
ANOVA	Age	0.38
Kruskal–Wallis	Treatment duration	0.53
Kruskal–Wallis	Symptom duration	0.81
Kruskal–Wallis	EDSS score	0.1
Kruskal–Wallis	Number of neurological hospitalizations	0.13
Fisher	Education	0.36
Fisher	Number of lesions on T2-weighted images	0.52
Fisher	Sex	0.35
Fisher	Marital status	0.19
Fisher	Livelihood	0.34
Fisher	Family	**0.01**
Fisher	Living with a family/alone	0.99
Fisher	Comorbidities	0.6
Fisher	Accidents and injuries	0.11
Fisher	Form of MS	0.87

**Table 8:** *p*-values of the tests evaluating correlations between clinically diagnosed mental disorders during the lifetime of patients and the specified variables and the corresponding values of correlation coefficients.

Test	Variables	*p*	Value of the Coefficient of the Correlation
Point-double series correlation coefficient	Age	0.46	−0.07
Point-double series correlation coefficient	Treatment duration	0.3	0.1
Point-double series correlation coefficient	Symptom duration	0.27	0.11
Point-double series correlation coefficient	EDSS score	**0.05**	0.19
Point-double series correlation coefficient	Number of neurological hospitalizations	0.31	0.1
Fisher	Education	0.79	
Fisher	Number of lesions on T2-weighted images	0.66	
ϕ Correlation coefficient	Sex	0.67	0.06
Chi-squared	Marital status	0.09	
Fisher	Livelihood	0.17	
ϕ Correlation coefficient	Family	0.35	0.11
ϕ Correlation coefficient	Living with a family/alone	0.76	0.03
ϕ Correlation coefficient	Comorbidities	0.54	0.08
ϕ Correlation coefficient	Accidents, injuries	1	0.03
Fisher	Form of MS	1	

## Data Availability

Data are at request to the corresponding author.

## Appendix A

**Table A1:** Distribution of the risk of suicide at the time of the assessment using the MINI Questionnaire based on comorbidities.

	No		Yes	
	*n*	*n* [%]	*n*	*n* [%]
Low	44	93.62	53	94.64
Medium	0	0	2	3.57
High	3	6.38	1	1.79

**Table A2:** Distribution of the risk of suicide at the time of the assessment using the MINI Questionnaire based on accidents and injuries.

	No		Yes	
	*n*	*n* [%]	*n*	*n* [%]
Low	93	93.94	4	100
Medium	2	2.02	0	0
High	4	4.04	0	0

**Table A3:** Distribution of the risk of suicide at the time of the assessment using the MINI Questionnaire based on the form of multiple sclerosis.

	Primary-Progressive		Relapsing–Remitting		Secondary-Progressive	
	*n*	*n* [%]	*n*	*n* [%]	*n*	*n* [%]
Low	2	66.67	85	95.51	10	90.91
Medium	0	0	2	2.25	0	0
High	1	33.33	2	2.25	1	9.09

**Table A4:** Descriptive statistics of the risk of suicide at the time of the assessment using the MINI Questionnaire based on the duration of MS treatment.

Risk of Suicide at the Time of the Assessment	*n*	Min	Max	Median	q1	q3	Mean	sd
Low	97	0	30	4	2	9	5.94	5.67
Medium	2	4	8	6	5	7	6	2.83
High	4	1	16	2.5	1	7	5.5	7.14

**Table A5:** Descriptive statistics of the risk of suicide at the time of the assessment using the MINI Questionnaire based on the duration of MS symptoms.

Risk of Suicide at the Time of the Assessment	*n*	Min	Max	Median	q1	q3	Mean	sd
Low	97	0	40	8	4	16	10.43	8.26
Medium	2	14	27	20.5	17.25	23.75	20.5	9.19
High	4	2	35	14	2	28.25	16.25	16.86

**Table A6:** Descriptive statistics of the risk of suicide at the time of the assessment using the MINI Questionnaire based on the EDSS scores.

Risk of Suicide at the Time of the Assessment	*n*	Min	Max	Median	q1	q3	Mean	sd
Low	97	0.5	8	3	2	4	3.15	1.6
Medium	2	2.5	3.3	2.9	2.7	3.1	2.9	0.57
High	4	2	6	5	3.5	6	4.5	1.92

**Table A7:** Descriptive statistics of the risk of suicide at the time of the assessment using the MINI Questionnaire based on the number of lesions on T2-weighted images.

Risk of Suicide at the Time of the Assessment	*n*	Min	Max	Median
Low	97	1	2	2
Medium	2	2	2	2
High	4	2	2	2

Description: 1—11–20, 2—>20.

**Table A8:** Descriptive statistics of the risk of suicide at the time of the assessment using the MINI Questionnaire based on the number of neurological hospitalizations.

Risk of Suicide at the Time of the Assessment	*n*	Min	Max	Median	q1	q3	Mean	sd
Low	97	1	50	2	1	4	4.76	7.73
Medium	2	1	10	5.5	3.25	7.75	5.5	6.36
High	4	2	6	3.5	2.75	4.5	3.75	1.71

**Table A9:** Distribution of psychiatric diagnoses at the time of the clinical assessment based on comorbidities.

	No		Yes	
	*n*	*n* [%]	*n*	*n* [%]
Not found	21	44.68	21	38.89
Adjustment disorders (F43.2)	5	10.64	6	11.11
Anxiety disorder (F41)	6	12.77	6	11.11
Depression (F32/F06)	8	17.02	17	31.48
Cognitive disorder (F06.7)	5	10.64	3	5.56
Bipolar affective disorder (F31)	2	4.26	1	1.85

**Table A10:** Distribution of psychiatric diagnoses at the time of the clinical assessment based on accidents and injuries obtained from patient history.

	No		Yes	
	*n*	*n* [%]	*n*	*n* [%]
Not found	41	42.27	1	25
Adjustment disorders (F43.2)	11	11.34	0	0
Anxiety disorder (F41)	10	10.31	2	50
Depression (F32/F06)	25	25.77	0	0
Cognitive disorder (F06.7)	7	7.22	1	25
Bipolar affective disorder (F31)	3	3.09	0	0

**Table A11:** Distribution of psychiatric diagnoses at the time of the clinical assessment based on the form of multiple sclerosis.

	Primary-Progressive		Relapsing–Remitting		Secondary-Progressive	
	*n*	*n* [%]	*n*	*n* [%]	*n*	*n* [%]
Not found	1	33.33	36	41.38	5	45.45
Adjustment disorders (F43.2)	0	0	10	11.49	1	9.09
Anxiety disorder (F41)	0	0	12	13.79	0	0
Depression (F32/F06)	2	66.67	19	21.84	4	36.36
Cognitive disorder (F06.7)	0	0	7	8.05	1	9.09
Bipolar affective disorder (F31)	0	0	3	3.45	0	0

**Table A12:** Distribution of psychiatric diagnoses at the time of the clinical assessment based on the number of lesions on T2-weighted images.

	11–20		>20	
	*n*	*n* [%]	*n*	*n* [%]
Not found	1	16.67	41	43.16
Adjustment disorders (F43.2)	1	16.67	10	10.53
Anxiety disorder (F41)	1	16.67	11	11.58
Depression (F32/F06)	2	33.33	23	24.21
Cognitive disorder (F06.7)	1	16.67	7	7.37
Bipolar affective disorder (F31)	0	0	3	3.16

**Table A13:** Descriptive statistics of psychiatric diagnoses at the time of the assessment based on age.

Diagnosis at the Time of the Assessment	*n*	Min	Max	Median	q1	q3	Mean	sd
Not found	42	23	66	43	34	53	43.33	12.22
Adjustment disorders (F43.2)	11	24	59	40	36.5	44	39.27	9.76
Anxiety disorder (F41)	12	20	63	47.5	38	55.25	44.75	14.7
Depression (F32/F06)	25	28	66	49	36	53	46.08	11.33
Cognitive disorder (F06.7)	8	27	60	40.5	35.5	47.25	41.62	11.48
Bipolar affective disorder (F31)	3	18	40	39	28.5	39.5	32.33	12.42

**Table A14:** Descriptive statistics of psychiatric diagnoses at the time of the assessment based on the duration of multiple sclerosis treatment.

Diagnosis at the Time of the Assessment	*n*	Min	Max	Median	q1	q3	Mean	sd
Not found	42	0	15	4	2	6.75	4.81	3.62
Adjustment disorders (F43.2)	11	1	11	7	2	9.5	6	4.22
Anxiety disorder (F41)	10	0	9	2	1.25	3.75	2.8	2.53
Depression (F32/F06)	25	1	28	4	1	13	7.44	7.42
Cognitive disorder (F06.7)	8	1	15	3	2.75	7	5.62	5.32
Bipolar affective disorder (F31)	3	1	10	8	4.5	9	6.33	4.73

**Table A15:** Descriptive statistics of psychiatric diagnoses at the time of the assessment based on the duration of MS symptoms.

Diagnoses at the Time of the Assessment	*n*	Min	Max	Median	q1	q3	Mean	sd
Not found	42	0	31	6.5	3.25	12.75	9.33	8.07
Adjustment disorders (F43.2)	11	1	40	10	4	17.5	12.64	11.42
Anxiety disorder (F41)	12	0	25	8	4.75	11.25	9.08	6.58
Depression (F32/F06)	25	1	35	13	4	18	12.76	9.83
Cognitive disorder (F06.7)	8	4	23	6.5	4.75	21	11.38	8.63
Bipolar affective disorder (F31)	3	3	21	20	11.5	20.5	14.67	10.12

**Table A16:** Descriptive statistics of psychiatric diagnoses at the time of the assessment based on the EDSS scores.

Diagnosis at the Time of the Assessment	*n*	Min	Max	Median	q1	q3	Mean	sd
Not found	42	0.5	7	2.5	2	3.5	2.73	1.51
Adjustment disorders (F43.2)	11	1	4.5	3	2.5	3.5	2.96	1.06
Anxiety disorder (F41)	12	1.5	4.5	4	2	4	3.25	1.08
Depression (F32/F06)	25	1.5	8	3.5	2.5	4.5	3.98	1.98
Cognitive disorder (F06.7)	8	1.5	6	3.25	2.5	5	3.62	1.55
Bipolar affective disorder (F31)	3	2	5.5	2.5	2.25	4	3.33	1.89

**Table A17:** Descriptive statistics of psychiatric diagnoses at the time of the assessment based on the number of neurological hospitalizations.

Diagnosis at the Time of the Assessment	*n*	Min	Max	Median	q1	q3	Mean	sd
Not found	37	1	7	2	1	3	2.22	1.48
Adjustment disorders (F43.2)	9	1	11	2	1	10	4.67	4.36
Anxiety disorder (F41)	10	1	4	2	2	2.75	2.4	0.97
Depression (F32/F06)	24	1	7	2	1	3	2.54	1.62
Cognitive disorder (F06.7)	8	1	12	2.5	1	6	4.5	4.75
Bipolar affective disorder (F31)	3	4	12	5	4.5	8.5	7	4.36

**Table A18:** Distribution of clinically confirmed/clinically unconfirmed mental disorders occurring during the lifetime of patients based on comorbidities.

	No		Yes	
	*n*	*n* [%]	*n*	*n* [%]
No	17	36.17	16	28.57
Yes	30	63.83	40	71.43

**Table A19:** Distribution of clinically confirmed/clinically unconfirmed mental disorders occurring during the lifetime of patients based on accidents and injuries.

	No		Yes	
	*n*	*n* [%]	*n*	*n* [%]
No	32	32.32	1	25
Yes	67	67.68	3	75

**Table A20:** Distribution of clinically confirmed/clinically unconfirmed mental disorders occurring during the lifetime of patients based on the form of multiple sclerosis.

	Primary-Progressive			Relapsing–Remitting		Secondary-Progressive		
	*n*	*n* [%]		*n*	*n* [%]	*n*		*n* [%]
No	1	33.33		29	32.58	3		27.27
Yes	2	66.67		60	67.42	8		72.73
Yes	3	60	13	61.9	27	72.97	27	67.5

**Table A21:** Distribution of clinically confirmed/clinically unconfirmed mental disorders occurring during the lifetime of patients based on the number of lesions on T2-weighted images.

	11–20		>20	
	*n*	*n* [%]	*n*	*n* [%]
No	1	16.67	32	32.99
Yes	5	83.33	65	67.01

**Table A22:** Descriptive statistics of clinically confirmed/clinically unconfirmed mental disorders occurring during the lifetime of patients based on age.

Mental Disorders	*n*	Min	Max	Median	q1	q3	Mean	sd
No	33	23	66	43	37	53	44.36	11.98
Yes	70	18	66	42.5	32	52	42.46	12.12

**Table A23:** Descriptive statistics of clinically confirmed/clinically unconfirmed mental disorders occurring during the lifetime of patients based on the duration of multiple sclerosis treatment.

Mental Disorders	*n*	Min	Max	Median	q1	q3	Mean	sd
No	33	0	15	4	2	7	5.08	3.89
Yes	70	0	30	4	2	9.75	6.32	6.28

**Table A24:** Descriptive statistics of clinically confirmed/clinically unconfirmed mental disorders occurring during the lifetime of patients based on the duration of multiple sclerosis symptoms.

Mental Disorders	*n*	Min	Max	Median	q1	q3	Mean	sd
No	33	0	31	6	4	12	9.46	8.61
Yes	70	0	40	10	4	17	11.51	8.8

**Table A25:** Descriptive statistics of clinically confirmed/clinically unconfirmed mental disorders occurring during the lifetime of patients based on the EDSS scores.

Mental Disorders	*n*	Min	Max	Median	q1	q3	Mean	sd
No	33	1	7	2.5	2	3.5	2.76	1.54
Yes	70	0.5	8	3	2.12	4	3.41	1.61

**Table A26:** Descriptive statistics of clinically confirmed/clinically unconfirmed mental disorders occurring during the lifetime of patients based on the number of neurological hospitalizations.

Mental Disorders	*n*	Min	Max	Median	q1	q3	Mean	sd
No	33	1	23	2	1	3	3.64	4.68
Yes	70	1	50	2.5	1.25	4.75	5.26	8.54
